# Which features of an outpatient treatment for COVID-19 would be most important for pandemic control? A modelling study

**DOI:** 10.1098/rsif.2021.0319

**Published:** 2021-09-29

**Authors:** Jean-Philippe Lanoix, Youcef Mammeri, Jean-Luc Schmit, Michel Lefranc

**Affiliations:** ^1^ AGIR UR4294, Jules Verne University of Picardie, Amiens, France; ^2^ GRECO, Jules Verne University of Picardie, Amiens, France; ^3^ Infectious Disease Department, Amiens-Picardie University Medical Center, Amiens, France; ^4^ Neurosurgery Department, Amiens-Picardie University Medical Center, Amiens, France; ^5^ LAMFA, CNRS UMR7352, Picardie Jules Verne University, Amiens, France

**Keywords:** COVID-19, proactive treatment, model, pandemic, outpatient

## Abstract

The global pandemic of coronavirus disease 2019 (COVID-19) has challenged healthcare systems worldwide. Lockdown, social distancing, and screening are thought to be the best means of stopping the virus from spreading and thus of preventing hospital capacity from being overloaded. However, it has also been suggested that effective outpatient treatment can control pandemics. We adapted a mathematical model of the beneficial effect of lockdown on viral transmission and used it to determine which characteristics of outpatient treatment would stop an epidemic. The data on confirmed cases, recovered cases, and deaths were collected from Santé Publique France. After defining components of the epidemic flow, we used a Morris global sensitivity analysis with a 10-level grid and 1000 trajectories to determine which of the treatment parameters had the largest effect. Treatment effectiveness was defined as a reduction in the patients' contagiousness. Early treatment initiation was associated with better disease control—as long as the treatment was highly effective. However, initiation of a treatment with a moderate effectiveness rate (5%) after the peak of the epidemic was still better than poor distancing (i.e. when compliance with social distancing rules was below 60%). Even though most of today's COVID-19 research is focused on inpatient treatment and vaccines, our results emphasize the potentially beneficial impact of even a moderately effective outpatient treatment on the current pandemic.

## 1. Introduction

The severe acute respiratory syndrome coronavirus 2 (SARS-CoV-2) has infected more than 192 million people worldwide (COVID-19) since January 2020, and more than 4 million of the latter have died from coronavirus disease 2019 (COVID-19) [1]. Although around 85% [2] of cases of COVID-19 are mild, severe forms of the disease necessitate hospital admission and (often) intensive care—placing severe strain on health structures and systems.

To limit the influx of patients and to avoid exceeding hospital capacity, lockdown, social distancing and screening strategies are thought to be the best means of stopping viral transmission. Indeed, several recent studies showed the effectiveness (but also the limitations) of these strategies [3–6].

Most of the ongoing therapeutic research is focused on developing vaccines, decreasing the severity of COVID-19 or treating severe forms of the disease requiring intensive care; by contrast, only 5% of registered clinical trials are looking at outpatients with COVID-19, and even fewer are focusing on how to reduce contagiousness in this context. The lack of studies of contagiousness is slowing attempts to reduce the spread of the virus at a time when outpatients should be the primary target of therapeutic research on COVID-19.

Mathematical modelling can predict the course of the COVID-19 pandemic as a function of various factors and strategies. Recently, we modelled the beneficial effect of lockdown on viral transmission [7]. Here, we used an adapted model, made of six nonlinear differential equations with delay, to determine which features of a drug treatment would have a greater effect than social distancing on pandemic control. This approach has been used to model the Ebola epidemic [8].

Nowadays, many mathematical models have been proposed to help control the epidemic. Most of them deal with non-pharmaceutical methods. For example, one widely relied upon (Flaxman *et al*. [9]), individual-based model, suggested how isolation and quarantine might mitigate the epidemic spread. Boulmezaoud [10] proposed alternate strict and moderate lockdown periods based on a discrete SIR model. Liu *et al*. [11] studied the impact of school closure and movement restrictions through a statistical analysis. Mammeri [12] proposed a spatially explicit model to measure localized restrictions. A review of models proposed for non-pharmaceutical interventions during the COVID-19 has been written by Perra [13].

Concerning pharmaceutical interventions, most of the models deal with the vaccination. In Dashtbali & Mirzaie [14], Giordano *et al*. [15], Moore *et al*. [16] or Xu *et al*. [17], vaccination is modelled in a new compartment of SIR-type by considering that susceptible individuals are vaccinated without delay.

To the best of our knowledge, the present work is the first to model the effects of various features of treatment on the progression of the pandemic. Our result showed that treatment start and compliance with treatment are the most important factors for reducing the transmission. Even a compliance with treatment as low as 25% with an effectiveness rate of 20% would be better than the current social distancing strategy. Minimal effectiveness was provided and may be of paramount importance to develop drugs for treating ambulatory patients with COVID-19.

## 2. Material and methods

### 2.1. Data on confirmed cases and deaths

The data on confirmed cases, recovered cases and deaths came from the publicly available COVID-19 dataset curated by Santé Publique France (https://www.data.gouv.fr/fr/organizations/sante-publique-france/). These anonymized data are collected from the public health authorities, and so ethical approval was not required.

### 2.2. The mathematical model

**Figure 1.**
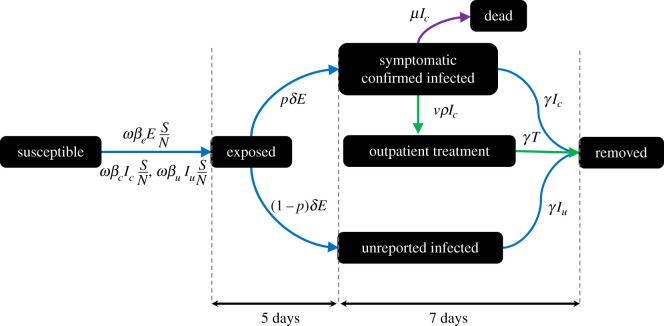
A compartmental representation of the model. Blue arrows describe the infection flow and green arrows describe the treatment. The purple arrow indicates death.

We focused on six components of the epidemic (figure 1): susceptible (*S*), a person who is at risk of contracting the disease; exposed (*E*), a person who has been exposed to the disease; symptomatic infected individual (*I*_c_), a confirmed positive case of infection with SARS-CoV-2 symptoms; outpatient treatment (*T*), a confirmed infected individual with mild symptoms and who can receive ambulatory treatment; unreported infected (*I*_*u*_), an asymptomatic or symptomatic infected individual who have not been reported; removed (*R*), a person whose disease has been cured and who is therefore removed from the model. The number of deaths due to the disease is denoted by D and satisfies D'(*t*) = *μI*_c_(*t*).

We first assumed that a susceptible individual can be infected by exposed individuals, confirmed symptomatic infected individuals and unreported infected individuals. We further assumed that only confirmed infected individuals with symptoms can be treated. The system is governed by six ordinary differential equations, as follows:

$$\left\{ \begin{array}{c} \begin{array}{ccc} S^{\prime }(t) & & \;=-\omega (t)(\beta_{e}E+\beta_{c}I_{c}+\beta_{u}I_{u})\frac{S}{N}\\ E^{\prime }(t) & & =\omega (t)(\beta_{e}E+\beta_{c}I_{c}+\beta_{u}I_{u})\frac{S}{N}-\delta E\\ I_{u}^{\prime }(t) & & \;=(1-p)\delta E-\gamma I_{u} \end{array}\;\\ \begin{array}{ccc} I_{c}^{\prime }(t) & & \;=p\delta E-(\gamma +\mu )I_{c}-\nu \rho I_{c}(t-\tau )\\ T^{\prime }(t) & & \;=\nu \rho I_{c}(t-\tau )-\gamma T\\ \;R^{\prime }(t) & & \;=\gamma (I_{u}+I_{c}+T). \end{array}\; \end{array}\right.$$

The model's parameters are described in electronic supplementary material, figure S1. The parameter *ω* is the average contact rate, *β*_*e*_, *β*_*c*_ and *β*_*u*_, are the probability transmission due to the exposed, confirmed infected, unreported infected individuals, respectively. The latency period is equal to 1/*δ*, while the recovery period is equal to 1/*γ*. The probability of being confirmed symptomatic is *p*, and the probability of being unreported is 1 − *p*. The death rate is denoted by *μ*.

Given that treatment is not instantaneous, a differential equation with *τ* is used to account for the time interval between treatment initiation and the onset of the drug's effectiveness. Here, *N* corresponds to the total living population. It is noteworthy that the mean number of contacts *ω* varies with time, in order to account for the start and end of lockdown.

Details about parameter calibration and model resolution can be found in the electronic supplementary material.

### 2.3. Treatment features

Four crucial parameters determine how effectively outpatient treatment can control an epidemic: the drug's effectiveness (*ν*, defined here as the ability to reduce a patient's contagiousness), the compliance with the treatment (*ρ*, defined here as the fraction of infected individuals who are compliant with treatment), its onset of action (*τ*, i.e. the time delay between a treatment's initiation and its effectiveness) and the treatment start date (*t*_start_) relative to the start of the epidemic.

We used a Morris global sensitivity analysis with a 10-level grid and 1000 trajectories to determine which of the treatment parameters had the largest effect [18]. The tested parameter ranges were 0 to 1 for the compliance with treatment *ρ*, 0.05 to 0.35 for the drug's effectiveness *ν*, 5 to 10 days for the time delay *τ*. The treatment start date *t*_start_ ranges from 52nd day to 198th day for the first simulation, i.e. from 16 March to 11 June. For the second simulation, the treatment start date *t*_start_ ranges from 52nd day to 163rd day again, i.e. from the 30 October to 17 March.

## 3. Results

### 3.1. The treatment initiation date

**Figure 2.**
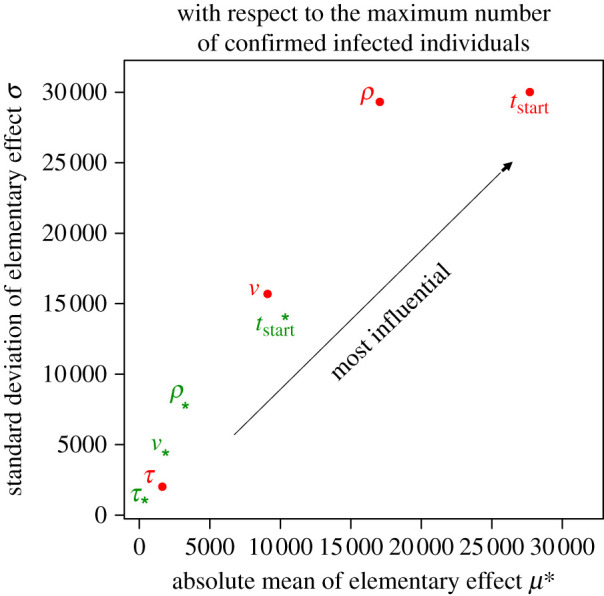
The treatment start date *t*_start_ is the most influential parameter regarding the maximum confirmed infected individuals, compliance with treatment *ρ* is also influential, and the onset of action *τ* and the drug's effectiveness *ν* are less influential. The absolute mean value μ* versus the standard deviation *σ* of elementary effects, with the maximum number of confirmed infected individuals as the output between 24 January and 8 September 2020 (green stars for the first lockdown period), and afterwards (red dots for the second lockdown period). The uppermost parameters (i.e. those with large *σ* values) correspond to nonlinear and interaction effects, while the rightmost parameters (i.e. those with large μ* values) correspond to linear and additive effects. The sensitivity analysis's output was the maximum confirmed infected individuals.

The most influential parameters (as judged by the absolute mean tendency (μ*) and the standard deviation (*σ*) of the elementary effects) are shown in figure 2. The further a parameter is from the origin, the more influential it is. The uppermost parameters (i.e. those with large *σ* values) correspond to nonlinear and interaction effects, while the rightmost parameters (i.e. those with large μ* values) correspond to linear and additive effects. The treatment start date (*t*_start_) had the greatest influence on the epidemic peak (green stars in figure 2); the earlier the treatment was initiated, the flatter the peak—even when the drug's effectiveness (*ν*) was low and/or its onset of action (*τ*) was long. Once the first wave had passed, the onset of action had less influence, and effectiveness became a more influential parameter. The compliance with treatment (*ρ*) became an important driver of the epidemic (red dots in figure 2).

### 3.2. Treatment versus infection multiplier

Here, we assumed that the treatment's onset of action was 5 days, and that the treatment had been initiated when the lockdowns had ended.

**Figure 3.**
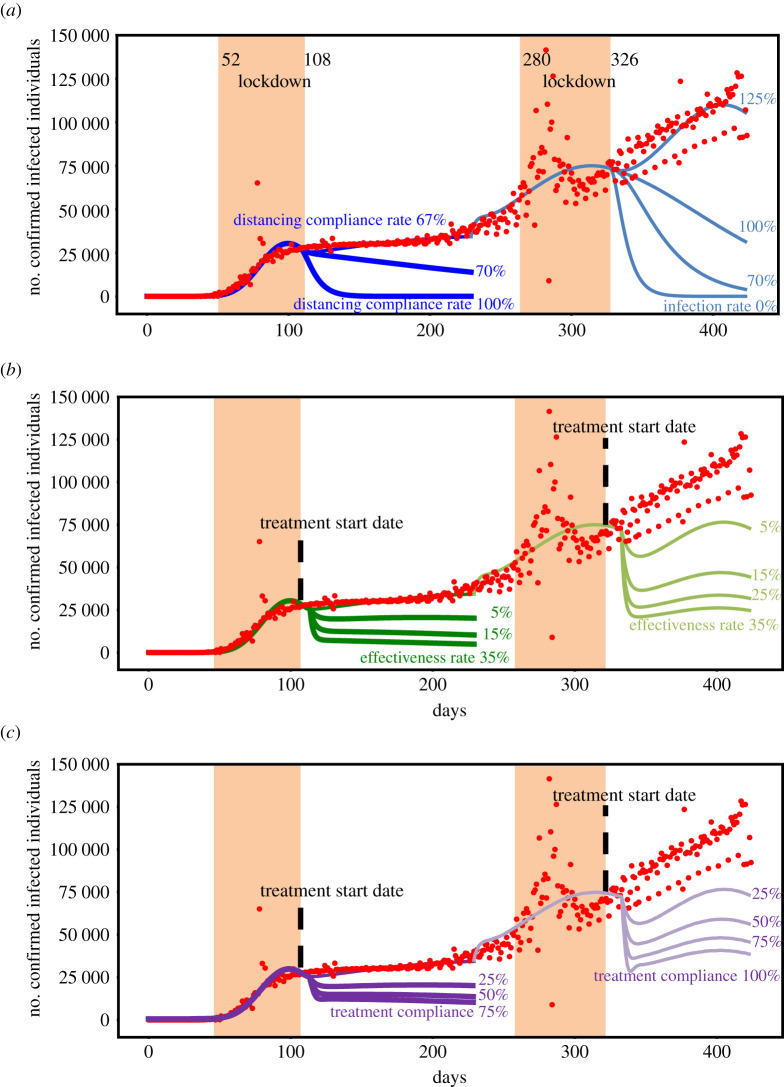
If the distancing compliance rate is 70% or less, treatment always offers better results even when the effectiveness rate is only 5%, or when the compliance rate is only 25%. (*a*) Comparisons of the number of confirmed symptomatic individuals (*I*c) as a function of the distancing compliance rate (from 67% to 100%) from the first lockdown end date (108th day) in dark blue, and as a function of the infection rate (from 0% to 125%) from the second lockdown end date (326th day) in light blue. Each blue curve represents a change in the rate. (*b*) Comparison of the number of confirmed infected individuals (*I*c) as a function of the treatment's effectiveness rate (from 5% to 35%, in 10% increments) when treatment starts the first lockdown end date (108th day) in dark green, and when treatment starts the second lockdown end date (326th day) in light green. Each green curve represents a change in the rate. (*c*) Comparison of the number of confirmed infected individuals (*I*c) as a function of the treatment's compliance rate (from 25% to 100%) when treatment starts the first lockdown end date (108th day) in dark purple, and when treatment starts the second lockdown end date (326th day) in light purple. Each purple curve represents a change in the rate. The red dots indicate the observed number of confirmed infected cases from Santé Publique France.

Concerning the time period 24 January 2020 to 8 September, we determined the number of confirmed infections as a function of compliance with social distancing rules (figure 3*a*), the treatment's effectiveness (figure 3*b*) and as a function of compliance with treatment (figure 3*c*). In mathematical terms, the drug's effectiveness *ν* is set to 0 for the absence of treatment, and the infection rate multiplier *η* varies between 0 and 0.36 (figure 3*a*). For treated patients with full compliance with treatment (*ρ* = 1), the drug's effectiveness *ν* varies between 0.05 and 0.35 (figure 3*b*). For treated patients with altered compliance with treatment, the drug's effectiveness *ν* is set to 0.2 and the compliance with treatment *ρ* varies between 0 and 1 (figure 3*c*). In both cases, the infection rate multiplier *η* is set to 0.36, which corresponds to the worst-calibrated scenario. It corresponds to 67% of compliance with the distancing rule.

We found that with an effectiveness rate of 16% 5 days after administration with full compliance with treatment, outpatient treatment is as good as a distancing compliance rate of 71%. For an effectiveness rate of 20% 5 days after administration, 79% compliance with treatment is as good as a distancing compliance rate of 71%. If the distancing compliance rate is 70% or less, treatment always offers better results—even when the effectiveness rate is only 5%, or when the compliance rate is only 25%. By way of an example, the number of confirmed infected cases in France at 8 September (the red dotted line in figure 3) corresponds to a compliance rate of 67%, and an effectiveness rate of only 5% flattens the epidemic curve in this scenario (figure 3*b*), as well as a compliance rate of 25% (figure 3*c*).

Concerning the time period 8 September 2020 to 20 March 2021, because of the emergence of new strains, we determined the number of confirmed infections as a function of infection multiplier (figure 3*a*), the treatment's effectiveness (figure 3*b*) and the compliance with treatment (figure 3*c*). In mathematical terms, the drug's effectiveness *ν* is set to 0 for the absence of treatment, and the infection rate multiplier *η* varies between 0 and 1.25 (figure 3*a*). For treated patients with full compliance with treatment (*ρ* = 1), the drug's effectiveness *ν* varies between 0.05 and 0.35 (figure 3*b*). For treated patients with altered compliance with treatment, the drug's effectiveness *ν* is set to 0.2 and the compliance with treatment *ρ* varies between 0 and 1 (figure 3*c*). In both cases, the infection rate multiplier *η* is set to 1.25, which corresponds to the worst-calibrated scenario.

We found that with an effectiveness rate of 16% 5 days after administration with full compliance with treatment, outpatient treatment is as good as reducing the infection transmission by 25%. For an effectiveness rate of 20% 5 days after administration, 79% compliance of treatment is as good as good as reducing the infection transmission by 25%. If the multiplier rate reduction is 24% or less, treatment always offers better results—even when the effectiveness rate is only 5%, or when the compliance rate is only 25. By way of an example, the number of confirmed infected cases in France on 20 March (the red dotted line in figure 3) corresponds to an increasing of 125% of the infection rate, and an effectiveness rate of only 5% flattens the epidemic curve in this scenario (figure 3*b*), as well as a compliance rate of 25% (figure 3*c*).

### 3.3. Optimal effectiveness

**Figure 4.**
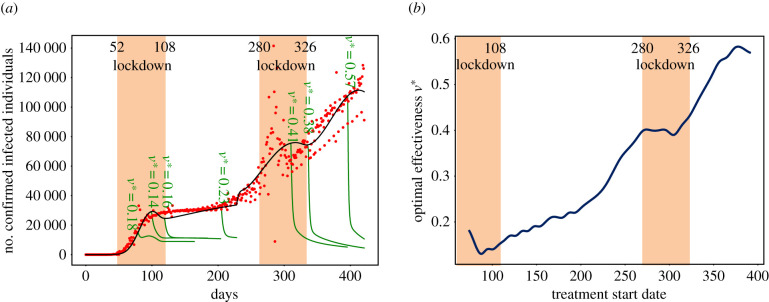
The earlier treatment starts, the less effective a treatment needs to be. A lower effectiveness is required when combining with lockdown. (*a*) Comparisons of the number of confirmed infected individuals (*I*c) as a function of the treatment's optimal effectiveness rate when treatment is initiated 74th, 93th, 103th, 200th, 296th, 321th or 382th day. Green curves represent changes in the optimal rate. (*b*) The optimal effectiveness rate ν* with respect to the start of treatment (from 74th day to 382th day). The optimal treatment effectiveness rates are required to reach 10 000 confirmed infected individuals *I*c, 30 days after the treatment is initiated.

We next sought to determine the minimum effectiveness rate (parameter minimums are symbolized with *) required to produce a target number of confirmed symptomatic infected individuals (*I*_c_*) at a given time t* (figure 4).

By way of an example, we assumed that the objective is set in the number of confirmed infected individuals as *I*_c_* = 10 000, 30 days after treatment initiation (which corresponds to more than a 10-fold reduction relative to the absence of treatment). Figure 4*a* shows that the relative decrease in the peak number of confirmed infected individuals depends on the treatment start date: the longer one waits to introduce treatment, the more effective that treatment needs to be (figure 4*b*). Indeed, a drug administered during the beginning of the epidemic could be less effective (an effectiveness rate of 18%, if treatment starts day 69, i.e. 30 days before the first epidemic peak), whereas nowadays it must be more effective (an effectiveness rate of 57% if treatment starts day 400).

A lower effectiveness rate is less problematic during the lockdowns. The rate goes from 18% to 14% during the first lockdown, and the rate goes from 41% to 38% during the second lockdown.

## 4. Discussion

To the best of our knowledge, the present study is the first to have determined which features of a candidate treatment have the greatest influence on stopping the COVID-19 pandemic. We showed that to reduce the number of infected individuals by a factor of 10 000, starting treatment early is the best option. We showed that lower effectiveness is required when combining with the lockdown. Our findings on minimal effectiveness may be of value for pharmaceutical companies repurposing old drugs or developing new drugs for treating ambulatory patients with COVID-19 during a putative ‘wave’.

Most importantly, our study's key finding is that a treatment with an effectiveness rate as low as 5% would be better than the current social distancing strategy (or rather the current rate of compliance with distancing rules) for reducing the transmission of COVID-19. Our result showed that compliance with treatment is one of the most important factors determining the success of reducing the transmission (figures 2 and 3). Even a compliance with treatment as low as 25% would be better than the current social distancing strategy. France were facing a plateau ‘wave’ or even a new ‘wave’. Social distancing rules are not sustainable, and new strains of the virus are emerging. Our model shows that a compliance rate of over 60% would have prevented a second wave; however, the value in France was gradually falling below that threshold. Hence, finding a treatment that would help to slow down—even a little—or stop the epidemic is imperative. These findings are likely to be of value to clinical trial investigators, who usually seek a clinically meaningful effect of at least 20% [19].

The concept of curative treatment of outpatients has not previously been applied to the control of infectious disease epidemics. For example, curative treatment of influenza has been reserved for symptomatic individuals, and prophylactic treatment has been reserved for at-risk patients (i.e. for non-infected asymptomatic patients) [20]. In cholera outbreaks, only hospitalized patients and those with moderate-to-severe disease are treated, and prophylaxis is not recommended.

However, three issues (safety, testing and follow-up) arise when considering the large-scale treatment of outpatients. First, the candidate drug(s) must be very safe in a population with no signs of disease. Given that the drug will be widely distributed, side effects must be very rare and non-serious; otherwise, the risk–benefit ratio will not be acceptable, and any potential epidemiological benefits will be lost. Second, this strategy requires large-scale testing and thus significant laboratory resources. However, the population is now accustomed to testing, and most countries have already implemented point-of-care screening. Furthermore, treatment after testing will probably be better received than the current self-isolation and sick leave measures. The introduction of rapid tests (less than 30 min) allows for wide expansion. Lastly, follow-up procedures must be easy to perform and reliable. The treatment should not require repeated blood tests, and the preferred use of telemedicine applications might help to avoid overloading the healthcare system. The lack of a need for follow-up would be better still because the patient would be rapidly and definitively cured.

The candidate drug's onset of action is an important epidemiological and medical parameter in the putative ambulatory treatment of patients with COVID-19—notably with regard to transmission control and potential disease aggravations [21]. We tested a value of between 5 and 10 days in our model. Although such a long onset of action would be unlikely, even then outpatient treatment would be very effective. Our model voluntarily underestimated treatment efficacy based on that parameter but nevertheless highlighted the treatment's ability to control the epidemic.

Delay differential equations are widely used in epidemiology in general and in the field of infectious disease in particular [22,23]. They have been used to describe the propagation of influenza, severe acute respiratory syndrome, hepatitis B virus and even COVID-19 [24–27]. However, the time interval in these models was related to the time delay for disease transmission or the latency period. Here, the time interval corresponded to a drug's onset of action.

Our study had several limitations. First, our comparison of social distancing and efficacy did not take account of uncertainties in compliance with distancing rules, e.g. behavioural shifts with regard to wearing masks. Second, the compliance with treatment could be more sophisticated to account patient's behaviour. Third, we assumed that all tested patients would be treated and did not calculate the number of patients to treat required to achieve control. However, our results prove that outpatient treatment must be a primary research focus if the epidemic is to be controlled. However, and since most of the patients arriving in the Amiens University Hospital are symptomatic, only confirmed individuals are treated in our model. As noted by Wiseman *et al*. [28], it would be interesting to be able to perform this very early treatment of exposed individuals.

The number of asymptomatic people with COVID-19 has been certainly largely underestimated [2,29]; if this is true, then the 24 million individuals infected since January 2020 represent the tip of the iceberg. Controlling the pandemic will require the treatment of a huge number of people. However, in view of the global economic crisis caused by SARS-CoV-2, ending the epidemic is essential.

## Data accessibility

Data are the publicly available COVID-19 dataset curated by Santé Publique France https://www.data.gouv.fr/fr/organizations/sante-publique-france/. These anonymized data are collected from the public health authorities, and so ethical approval was not required.
